# Clinical Manifestations, Long-Term Trends, and Risk Factors for Treatment Failure in Native Vertebral Osteomyelitis: A 26-Year Mayo Clinic Experience

**DOI:** 10.1093/cid/ciag048

**Published:** 2026-02-04

**Authors:** Takahiro Matsuo, Fabio Borgonovo, Brian D Lahr, Francesco Petri, Rita Igwilo-Alaneme, Sergio L Alvarez Mulett, Seyed Mohammad Amin Alavi, Doug W Challener, Ahmad Nassr, Paul M Huddleston, Aaron J Tande, Elie F Berbari

**Affiliations:** Department of Medicine, Division of Public Health, Infectious Diseases, and Occupational Medicine, Mayo Clinic, Rochester, Minnesota, USA; Department of Medicine, Division of Public Health, Infectious Diseases, and Occupational Medicine, Mayo Clinic, Rochester, Minnesota, USA; Department of Infectious Diseases, “Luigi Sacco” University Hospital, Milan, Italy; Department of Biomedical Statistics and Informatics, Mayo Clinic, Rochester, Minnesota, USA; Department of Medicine, Division of Public Health, Infectious Diseases, and Occupational Medicine, Mayo Clinic, Rochester, Minnesota, USA; Department of Infectious Diseases, “Luigi Sacco” University Hospital, Milan, Italy; Department of Medicine, Division of Public Health, Infectious Diseases, and Occupational Medicine, Mayo Clinic, Rochester, Minnesota, USA; Department of Medicine, Division of Public Health, Infectious Diseases, and Occupational Medicine, Mayo Clinic, Rochester, Minnesota, USA; Faculty of Medicine, Ahvaz Jundishapur University of Medical Sciences, Ahvaz, Iran; Department of Medicine, Division of Public Health, Infectious Diseases, and Occupational Medicine, Mayo Clinic, Rochester, Minnesota, USA; Department of Orthopedic Surgery, Mayo Clinic, Rochester, Minnesota, USA; Department of Orthopedic Surgery, Mayo Clinic, Rochester, Minnesota, USA; Department of Medicine, Division of Public Health, Infectious Diseases, and Occupational Medicine, Mayo Clinic, Rochester, Minnesota, USA; Department of Medicine, Division of Public Health, Infectious Diseases, and Occupational Medicine, Mayo Clinic, Rochester, Minnesota, USA

**Keywords:** native vertebral osteomyelitis (NVO), treatment failure, risk factors, trend analysis, *Staphylococcus aureus*

## Abstract

**Background:**

Native vertebral osteomyelitis (NVO) is a life-threatening spinal infection with rising incidence and significant morbidity. Despite its growing burden, long-term data on clinical characteristics, management trends, and outcomes remain limited.

**Methods:**

We conducted a 26-year multicenter retrospective cohort study of adults (≥18 years) diagnosed with NVO at Mayo Clinic sites between 1999 and 2024. Demographic, microbiologic, treatment, and outcome data were analyzed across five time periods. Predictors of treatment failure were assessed using a multivariable competing risk model.

**Results:**

Among 1255 patients (median age 67; 66% male), lumbosacral involvement was most common (65%), and 21% had multilevel involvement. Pathogens were identified in 77%, most commonly *Staphylococcus aureus* (49%; Methicillin-susceptible *S. aureus* 37%, methicillin-resistant *S. aureus* 13%). Over time from 1999–2004 to 2020–2024, Gram-negative bacilli increased from 6% to 14% (*P* = .048). Comorbidities including chronic kidney disease (10% to 21%), active chemotherapy (6% to 11%), and immunosuppression (8% to 17%) increased significantly. Additionally, 1-year treatment failure declined (16% to 10%). In multivariable analysis, diabetes mellitus (subdistribution hazard ratio [sHR] 1.92, 95% CI 1.18–3.13) and multilevel involvement (sHR 1.67, 95% CI 1.17–2.38) were associated with increased incidence of treatment failure, while concurrent infections (sHR 0.57, 95% CI 0.37–0.87) and higher Charlson Comorbidity Index (sHR 0.62, 95% CI 0.43–0.90) were associated with lower failure.

**Conclusions:**

This large multicenter cohort highlights increasing host complexity, shifting microbiology, and predictors of failure, emphasizing the importance of early risk stratification and tailored strategies, such as multidisciplinary evaluation and close follow-up of high-risk patients to improve outcomes.

## Teaser

This 26-year multicenter study of 1255 NVO patients reveals increasing comorbidities, shifting microbiology, and key predictors of failure and mortality, underscoring the complexity of NVO and the need for tailored management strategies in high-risk populations.

## Graphical Abstract

**Graphical Abstract:**
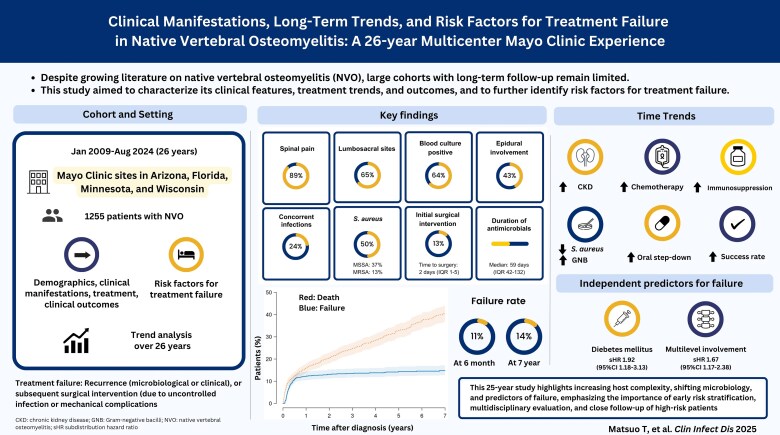

**(See the Editorial Commentary by Zimmerli on pages e1181–3.)**

Native vertebral osteomyelitis (NVO) is a serious, life-threatening, spinal infection with significant morbidity, accounting for 3–6% of all osteomyelitis cases [1, 2]. It can lead to neurologic deficits, spinal instability, and persistent pain, often requiring prolonged hospitalizations, surgical interventions, and long-term disability [3]. Mortality rates may reach 21% [4–6], and a significant proportion of survivors experience functional impairment and reduced quality of life [3–5, 7]. Timely diagnosis and appropriate therapy are therefore essential to avoid adverse outcomes.

Over the past three decades, NVO incidence has increased due to an aging population, increased comorbidities, and improved diagnostics [2, 8–11]. The incidence in Germany is projected to rise from 12.4 per 100,000 in 2019 to 21.5 by 2040, especially among adults ≥75 years [11]. Despite growing recognition of its burden, the long-term epidemiology of NVO—particularly shifts in host demographics, microbiology, and prognostic factors—remains poorly characterized.

Clinical outcomes in NVO remain variable. Reported treatment failure rates range from 10%–48% [12–15] and are frequently associated with long-term morbidity [7, 16]. The landmark French randomized controlled trial (RCT) [17] shaped the latest Infectious Diseases Society of America (IDSA) guideline recommending a minimum 6-week antibiotic course [3]. Subsequent observational studies have identified potential risk factors for failure [12–14, 18, 19]. Although these studies have provided valuable insights, findings have varied, often limited by small sample sizes, heterogeneous populations, and short follow-up. Consequently, high-risk patients remain partially identified, and robust evidence to guide clinical risk stratification is lacking.

To address these gaps, we conducted a 26-year multicenter cohort study of NVO. Our aims were to (1) characterize clinical features, treatment, and outcomes in a large contemporary cohort; (2) analyze temporal changes in patient demographics, microbiology, and management; and (3) identify independent predictors of treatment failure to inform modern clinical decision-making.

## METHODS

### Study Design and Setting

We conducted a multicenter retrospective cohort study of adults (≥18 years) diagnosed with NVO across Mayo Clinic sites in Minnesota, Arizona, Florida, and Wisconsin between 1 January 1999, and 31 August 2024. The Mayo Clinic Institutional Review Board approved the study (IRB# 24-010081). Patients with magnetic resonance imaging (MRI) findings consistent with NVO were identified from radiology report queries (Supplementary Methods 1). An infectious diseases physician confirmed eligibility using predefined diagnostic criteria: microbiological evidence from blood or spinal tissue cultures, broad range 16S ribosomal RNA polymerase chain reaction (PCR) on bone biopsy when available, histopathologic inflammation, or clinician-judged compatibility based on symptoms (eg, back pain, fever, or neurologic deficits) [7, 12, 18, 20]. Blood cultures were considered diagnostic only when isolates represented recognized pathogens. Organisms typically considered contaminants (eg, coagulase-negative staphylococci, *Cutibacterium acnes*) required tissue isolation or repeated positive blood cultures with consistent clinical and imaging findings. In addition, non–culture-based diagnostic tests (eg, serology, histopathology, or pathogen-specific PCR) were performed when clinically indicated for atypical organisms. Exclusion included noninfectious etiologies (eg, malignancy, inflammatory disease), spinal hardware or surgical site infections, decubitus ulcer-related infections, missing essential data from external referral, or lack of research authorization per Minnesota regulations. Only the first episode per patient was included.

Data were abstracted into REDCap, a secure Mayo Clinic platform [21], including demographics, comorbidities, clinical features, diagnostics, antimicrobial therapy, surgeries, and outcomes. Patients were followed until treatment failure, death, or loss to follow-up.

### Definitions

Treatment failure was defined as either recurrence of infection or the need for subsequent surgical intervention. Recurrence was defined as NVO occurring after completion of initial treatment and classified as either microbiological recurrence, with pathogen(s) identified, or clinical recurrence, with consistent signs and symptoms of NVO but no pathogen(s) identified [7, 12, 18]. Surgical interventions were considered treatment failure if they were required due to uncontrolled infection after at least four weeks of antimicrobial therapy, or due to mechanical complications such as instability or deformity [7], considering their significant impact on patient functional outcomes. Surgical interventions within the first four weeks of treatment were considered part of the initial management rather than treatment failure. Epidural, paravertebral, and psoas involvement were defined as the presence of enhancement, phlegmon, or abscess on MRI, due to challenges in clearly distinguishing these findings [7]. Concurrent infections were defined as infection sites outside of epidural, paraspinal, or psoas involvement, including infective endocarditis, other intravascular infections, septic arthritis, prosthetic joint infections, and septic emboli within 30 days of NVO diagnosis. Immunosuppressive condition was defined as any of the following: corticosteroid use equivalent to more than 20 mg of prednisone daily for over 2 weeks; use of other immunosuppressive agents; active hematologic or solid malignancy; human immunodeficiency virus (HIV)/AIDS; primary immunodeficiency; or a history of solid organ or hematopoietic stem cell transplantation. Multilevel involvement was defined as an infection involving more than two vertebral bodies.

### Statistical Analysis

Baseline characteristics were described by quartiles for continuous variables and frequencies for discrete variables. Outcomes were described by cumulative event rates over follow-up, calculated using either the cumulative incidence function estimator (for treatment failure, accounting for competing risk of death), or the Kaplan–Meier method (for mortality). To elucidate the clinical factors that influence treatment failure, we conducted a multivariable competing risks analysis based on the Fine and Gray subdistribution hazard model that considered failure as the primary event and death as the competing event. The prespecified potential predictors included 10 baseline covariates (age, sex, diabetes, Charlson Comorbidity Index [CCI], end-stage renal disease, immunosuppressed status, concurrent infections, multi-level involvement, spinal-associated abscess, pathogen) and 2 time-dependent covariates (total duration of antibiotics, surgical intervention). The clinical determinants of mortality were analyzed in a similar multivariable fashion via Cox proportional hazards regression, with treatment failure incorporated into the model as an additional time-dependent covariate. All analyses were done in R version 4.4.1. A more detailed description of these statistical methods can be found in the Supplementary Methods 2.

## RESULTS

### Patient Demographics and Clinical Manifestations

Among 1825 patients identified with suspected NVO, 570 were excluded: noninfectious etiologies (n = 169), spinal hardware–associated infections (n = 152), surgical site infections (n = 85), decubitus ulcer–related infections (n = 7), missing outside data (n = 77), or lack of Minnesota research authorization (n = 80). The remaining 1255 patients met the NVO definition. The median age was 67 years (interquartile range [IQR], 57–75); 66% were male, and 93% were White. The median CCI was 3 (IQR, 2–6); common comorbidities included diabetes mellitus (21%) and chronic kidney disease (CKD) (17%). Intravenous (IV) drug use was rare (2%). Additional demographic are shown in Table 1.

**Table 1.** Demographics of 1255 Patients With NVO

Characteristic	N	Overall (N = 1255)
Age, y	1255	67 (57–75)
Male sex	1255	828 (66%)
Body mass index, kg/m²	1214	28 (24–34)
Race	1213	…
American Indian/Alaskan Native	…	15 (1%)
Asian	…	20 (2%)
Black	…	31 (3%)
White	…	1132 (93%)
Other	…	15 (1%)
Ethnicity: Hispanic or Latino	1121	35 (3%)
Diabetes mellitus	1255	265 (21%)
Chronic kidney disease	1255	209 (17%)
Hemodialysis	1255	20 (2%)
Chronic liver disease	1255	…
None	…	1155 (92%)
Mild	…	62 (5%)
Moderate/severe	…	38 (3%)
Chronic pulmonary disease	1255	169 (13%)
Congestive heart failure	1255	152 (12%)
Myocardial infarction	1255	64 (5%)
Rheumatologic disease	1255	62 (5%)
Peripheral vascular disease	1255	197 (16%)
Cerebrovascular disease	1255	88 (7%)
Metastatic solid tumor	1255	50 (4%)
Other cancer	1255	192 (15%)
Active chemotherapy^a^	1255	94 (7%)
Charlson Comorbidity Index	1255	3 (2–6)
IV drug user	1255	23 (2%)
History of any transplant	1255	40 (3%)
Bone marrow transplant	40	17 (42%)
Solid organ transplant	40	23 (58%)
Immunosuppressive condition^b^	1255	183 (15%)

Data are presented as median (25th percentile–75th percentile) for continuous variables and number (percentage) of patients for categorical variables. N represents the number of non-missing values.

Abbreviation: IV, intravenous; NVO, native vertebral osteomyelitis.

^a^Received within 90 d of NVO diagnosis.

^b^Defined as any of the following: corticosteroid use equivalent to more than 20 mg of prednisone daily for over 2 wks; use of other immunosuppressive agents; active hematologic or solid malignancy; human immunodeficiency virus (HIV)/ AIDS; primary immunodeficiency; or a history of solid organ or hematopoietic stem cell transplantation.

Back pain was the most frequent symptom (89%), followed by fever (53%), motor weakness (38%), and numbness (27%). Lumbosacral involvement was most common (65%), followed by thoracic (38%) and cervical (13%) regions; 21% had multilevel involvement. Epidural involvement occurred in 43%, paravertebral in 42%, and psoas in 20%. Concurrent infections were identified in 24%, including infective endocarditis in 11% (Table 2).

**Table 2.** Clinical Manifestations of NVO

Characteristic	N	Overall (N = 1255)
Back pain	1255	1115 (89%)
Fever	1255	671 (53%)
Motor weakness	1255	482 (38%)
Numbness	1255	341 (27%)
WBC at diagnosis, ×10³/μL	1123	9 (7–12)
CRP at diagnosis, mg/dL	979	58 (16–141)
ESR at diagnosis, mm/h	837	60 (34–91)
Infected site^a^	1255	…
Cervical involvement	…	157 (13%)
Thoracic involvement	…	479 (38%)
Lumbosacral involvement	…	822 (65%)
Multilevel involvement	…	258 (21%)
Epidural involvement	1255	536 (43%)
Paravertebral involvement	1255	530 (42%)
Psoas involvement	1255	256 (20%)
Concurrent infections	1255	303 (24%)
Concurrent infections types^a^	303	…
Endocarditis	…	141 (47%)
Other intravascular infection	…	27 (9%)
Septic arthritis	…	62 (20%)
Prosthetic joint infection	…	21 (7%)
Pulmonary emboli or empyema	…	20 (7%)
CNS involvement^b^	…	35 (12%)
Others	…	46 (15%)
Polymicrobial pathogens	961	52 (5%)

Data are presented as median (25th percentile–75th percentile) for continuous variables and number (percentage) of patients for categorical variables. N represents the number of non-missing values.

Abbreviations: CNS, central nervous system; CRP, C-reactive protein; ESR, erythrocyte sedimentation rate; NVO, native vertebral osteomyelitis; WBC, white blood cell count.

^a^The possible choices listed below this variable are not mutually exclusive (ie, patients sometimes had multiple choices indicated), resulting in column totals >100%.

^b^CNS involvement includes brain emboli, abscess, and meningitis.

### Diagnosis and Microbiology

Blood cultures were obtained in 1180 (94%), with 759 (64%) positive. Bone biopsy was performed in 388 (31%), with positive tissue cultures in 241 (62%). Broad-range PCR was performed in 158 (13%) and was positive in 49 (31%), including 33 (67%) culture-negative cases. When both blood and tissue cultures were obtained, diagnostic yield increased to 76%, and the addition of PCR further improved diagnostic detection (77%). Overall, a causative pathogen was identified in 961 (77%) patients (Table 3).

**Table 3.** Diagnostic Methods and Microbiological Yield in NVO (N = 1255)

Diagnostic Methods	Cases	Result (n, %)
Blood culture obtained (all)	1255	1180 (94%)
Positive results	1180	759 (64%)
Blood culture only obtained (no tissue culture obtained)	1255	726 (58%)
Positive results	726	588 (81%)
Tissue culture obtained (all)^a^	1255	388 (31%)
Positive results	388	241 (62%)
Blood culture +/tissue culture +	241	81 (34%)
Blood culture −/tissue culture +	241	160 (66%)
Broad range PCR obtained (all)	388	158 (41%)
Positive results	158	49 (31%)
Tissue culture +/broad-range PCR +	49	16 (33%)
Tissue culture −/broad-range PCR +	49	33 (67%)
Combined blood + tissue culture obtained	1255	307 (24%)
Positive results^b^	307	233 (76%)
Combined blood + tissue culture + broad-range PCR obtained	1255	56 (4%)
Positive results^c^	56	43 (77%)
Pathogen identified	1255	961 (77%)

Abbreviations: CT, computed tomography; NVO, native vertebral osteomyelitis; PCR, polymerase chain reaction.

^a^CT-guided biopsy (n = 309), open biopsy (n = 79).

^b^Positive results indicate detection by either blood culture or tissue culture.

^c^Positive results indicate detection by any of the three methods (blood culture, tissue culture, or broad range PCR).

Methicillin-susceptible *Staphylococcus aureus* (MSSA; 37%) and methicillin-resistant *S. aureus* (MRSA; 13%) were the most common organisms, followed by viridans group streptococci (11%) and *Enterococcus faecalis* (7.3%). Gram-negative organisms accounted for approximately 13%, most frequently *Escherichia coli* (5%) and *Pseudomonas aeruginosa* (1.8%). Polymicrobial infections were identified in 52 patients (5%). Detailed microbiology is shown in Table 4.

**Table 4.** Identified Pathogens Among 961 Patients With NVO (1022 Total Pathogens)

Identified Pathogens^a^	Overall (N = 961)
Gram-positive bacteria	…
*Staphylococcus aureus*, methicillin-susceptible	351 (37%)
*Staphylococcus aureus*, methicillin-resistant	123 (13%)
Viridans group streptococci	103 (11%)
*Enterococcus faecalis*	70 (7.3%)
β-hemolytic streptococci	69 (7.2%)
Other *Streptococcus* spp.	31 (3.2%)
Coagulase-negative staphylococci	18 (1.9%)
*Streptococcus gallolyticus*	15 (1.6%)
*Cutibacterium acnes*	13 (1.4%)
*Staphylococcus lugdunensis*	7 (0.7%)
*Enterococcus faecium*	7 (0.7%)
*Enterococcus casseliflavus*	1 (0.1%)
*Nocardia* spp.	1 (0.1%)
Gram-negative bacteria	…
*Escherichia coli*	48 (5.0%)
*Pseudomonas aeruginosa*	18 (1.8%)
*Klebsiella* spp.	13 (1.4%)
*Enterobacter* spp.	10 (1.0%)
HACEK	6 (0.6%)
*Proteus* spp.	5 (0.5%)
*Serratia* spp.	3 (0.3%)
*Salmonella* spp.	1 (0.1%)
*Yersinia* spp.	1 (0.1%)
*Acinetobacter* spp.	1 (0.1%)
*Stenotrophomonas maltophilia*	1 (0.1%)
*Ralstonia* spp.	1 (0.1%)
*Pasteurella multocida*	1 (0.1%)
*Neiserria* spp.	1 (0.1%)
Anaerobe	38 (4.0%)
Others/atypical bacteria^b^	…
*Coxiella burnetii*	3 (0.3%)
*Brucella* spp	2 (0.2%)
*Candida* spp.	21 (2.2%)
*Coccidioides* spp.	10 (1.0%)
*Aspergillus* spp.	4 (0.4%)
*Scedosporium* spp.	1 (0.1%)
*Mycobacterium* spp.	19 (2.0%)

Abbreviations: HACEK, *Haemophilus, Aggregatibacter, Cardiobacterium, Eikenella,* and *Kingella* spp; NVO, native vertebral osteomyelitis.

^a^Fifty-two patients (5%) had polymicrobial infections.

^b^Some atypical pathogens listed in this table were identified by non–culture-based diagnostic methods (such as serology, histopathology, or pathogen-specific PCR).

### Treatment

IV antibiotic therapy was administered in 96% of patients (median 42 days [IQR, 42–60)] and oral antibiotics were used in 42% (median 110 days [IQR, 42–669]). The median total duration of therapy was 59 days (IQR, 42–132).

Surgery was performed in 13% of patients within a median of 2 days of NVO diagnosis (IQR, 1–5), most commonly posterior debridement with or without laminectomy (69%).

### Treatment Outcomes

**Figure 1.**
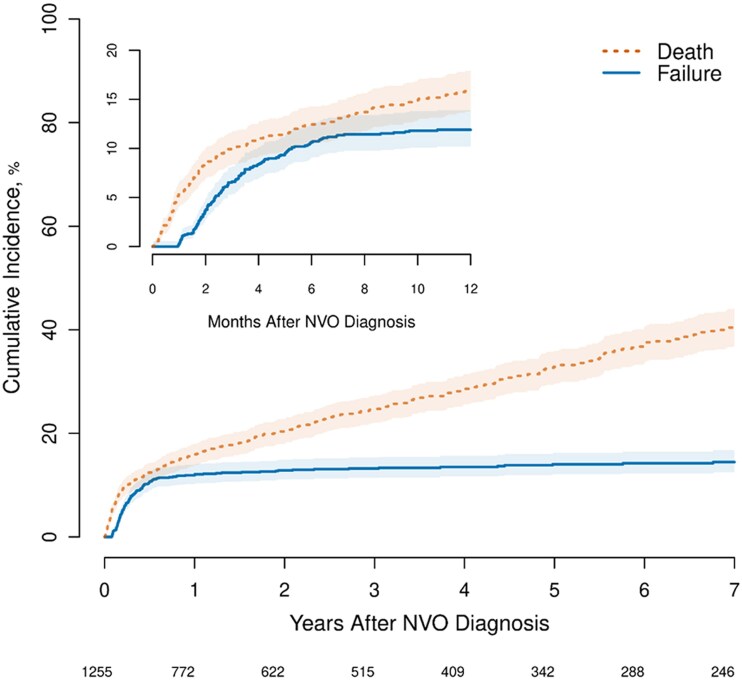
Cumulative incidence of treatment failure and all-cause mortality in patients with native vertebral osteomyelitis. The main panel shows long-term cumulative incidence curves up to 7 y after NVO diagnosis for treatment failure (solid blue line) and all-cause mortality (dotted orange line). The treatment failure curve accounts for the competing risk of death. The mortality curve reflects cumulative death irrespective of treatment failure. The inset in the upper left displays the cumulative incidence within the first 12 m to highlight early event dynamics. The number of patients at risk at each time point is shown below the *x*-axis. Abbreviation: NVO, native vertebral osteomyelitis.

During a median follow-up of 3.9 years (IQR, 1.6–9.5), 162 patients experienced treatment failure: 100 recurrences (64 clinical, 36 microbiological), 47 surgeries for uncontrolled infection, and 15 surgeries for mechanical reasons (including 7 biopsies, all culture-negative). Accounting for the competing risk of death, cumulative incidence of treatment failure at 6 months, 1 year, and 7 years was 11%, 12%, and 14%, respectively (Table 5). A total of 443 patients died during follow-up (59 after treatment failure and 384 without prior failure), corresponding to cumulative mortality rates of 12%, 16%, and 41% at the same time points. Cumulative incidence curves for treatment failure and mortality are shown in Figure 1.

**Table 5.** Treatment and Outcomes of NVO

Treatment and Outcomes	N	Overall (N = 1255)
Intravenous antibiotic use	1255	1206 (96%)
Duration of IV antibiotics, d	1206	42 (42–60)
Oral antibiotic use	1255	526 (42%)
Duration of PO antibiotics, d	526	110 (42–669)
Duration of total antibiotics, d	1255	59 (42–132)
Initial surgical intervention performed	1255	164 (13%)
Time to initial surgical intervention, d	164	2 (1–5)
Type of initial surgery	164	…
Posterior debridement with or without laminectomy	…	113 (69%)
Posterior fusion with implant placement	…	36 (22%)
Anterior debridement	…	11 (7%)
Anterior fusion without implant placement	…	6 (4%)
Anterior fusion with implant placement	…	2 (1%)
Anterior fusion with posterior implant placement	…	2 (1%)
Treatment failure	1255	…
No. of patients with events	…	162
Cumulative event rate—% (95% CI)^a^	…	…
At 3 m	…	7% (5%–8%)
At 6 m	…	11% (9%–12%)
At 9 m	…	12% (10%–13%)
At 1 y	…	12% (10%–14%)
At 2 y	…	13% (11%–15%)
At 3 y	…	13% (11%–15%)
At 5 y	…	14% (12%–16%)
At 7 y	…	14% (12%–17%)
Type of failure	162	…
Recurrence of infection	…	100 (62%)
Microbiological recurrence	…	36 (22%)
Clinical recurrence	…	64 (40%)
Subsequent surgical intervention for uncontrolled infection	…	47 (29%)
Surgical intervention due to mechanical reasons	…	15 (9%)
Death from any cause	1255	…
No. of patients with events	…	443
Cumulative event rate—% (95% CI)^a^	…	…
At 3 m	…	10% (8%–12%)
At 6 m	…	12% (11%–14%)
At 9 m	…	14% (12%–16%)
At 1 y	…	16% (14%–18%)
At 2 y	…	20% (18%–23%)
At 3 y	…	25% (22%–27%)
At 5 y	…	33% (30%–36%)
At 7 y	…	41% (37%–44%)

Abbreviations: CI, confidence interval; IV, intravenous; NVO, native vertebral osteomyelitis; PO, per os (oral).

^a^Cumulative event rates were calculated using a nonparametric cumulative incidence function estimator for treatment failure (accounting for competing risk of death) and using the Kaplan–Meier estimator for death.

### Changes in Baseline and Clinical Characteristics for Different Time Periods

The annual number of NVO cases increased across the 26-year study period, rising from 18 cases/year before 2005 to 99 cases/year after 2019. Changes in patient characteristics, management, and outcomes across five different time periods are summarized in Table 6. For brevity, we illustrate these changes below using descriptive statistics only from the first (1999–2004) and last (2020–2024) periods, although the reported trend tests are comprehensive.

**Table 6.** Changes in Baseline and Clinical Characteristics for Different Time Periods

Outcome	1999–2004 (N = 106)	2005–2009 (N = 144)	2010–2014 (N = 197)	2015–2019 (N = 314)	2020–2024 (N = 494)	*P*
Age, y	69 (54–75)	66 (55–74)	66 (56–77)	66 (57–75)	67 (57–76)	.170
Male sex	66 (62%)	90 (62%)	120 (61%)	210 (67%)	342 (69%)	.029
Body mass index, kg/m² (N = 1214)	28 (24–32)	27 (24–32)	29 (24–34)	29 (25–33)	29 (24–34)	.320
Race: White (N = 1213)	81 (95%)	132 (97%)	183 (94%)	293 (94%)	443 (91%)	.012
Diabetes mellitus	15 (14%)	21 (15%)	46 (23%)	75 (24%)	108 (22%)	.033
Chronic kidney disease	11 (10%)	13 (9%)	22 (11%)	57 (18%)	106 (21%)	<.001
Hemodialysis	1 (1%)	0 (0%)	0 (0%)	0 (0%)	19 (4%)	<.001
Cancer history	13 (12%)	17 (12%)	26 (13%)	51 (16%)	89 (18%)	.023
Active chemotherapy	6 (6%)	7 (5%)	7 (4%)	18 (6%)	56 (11%)	<.001
Charlson Comorbidity Index	3 (1–5)	3 (1–5)	3 (2–5)	3 (2–6)	4 (2–7)	<.001
IV drug user	3 (3%)	0 (0%)	3 (2%)	8 (3%)	9 (2%)	.668
History of any transplant	2 (2%)	4 (3%)	4 (2%)	13 (4%)	17 (3%)	.277
Immunosuppressive condition	9 (8%)	19 (13%)	23 (12%)	47 (15%)	85 (17%)	.010
Back pain	86 (81%)	132 (92%)	171 (87%)	264 (84%)	462 (94%)	.003
Fever	32 (30%)	93 (65%)	104 (53%)	169 (54%)	273 (55%)	.024
Motor weakness	27 (25%)	70 (49%)	78 (40%)	108 (34%)	199 (40%)	.407
Numbness	15 (14%)	54 (38%)	53 (27%)	72 (23%)	147 (30%)	.226
WBC at diagnosis (N = 1123)	8 (6–10)	9 (7–12)	9 (7–12)	9 (7–12)	9 (7–12)	.490
CRP at diagnosis (N = 979)	6 (1–12)	23 (6–84)	75 (21–148)	73 (29–175)	68 (26–143)	<.001
ESR at diagnosis (N = 837)	54 (24–82)	50 (33–81)	70 (39–100)	61 (40–90)	60 (34–97)	.050
Cervical involvement	11 (10%)	17 (12%)	27 (14%)	45 (14%)	57 (12%)	.914
Thoracic involvement	32 (30%)	47 (33%)	86 (44%)	119 (38%)	195 (39%)	.101
Lumbosacral involvement	64 (60%)	85 (59%)	124 (63%)	215 (68%)	334 (68%)	.022
Multilevel involvement	13 (12%)	23 (16%)	52 (26%)	86 (27%)	84 (17%)	.574
Epidural involvement	38 (36%)	53 (37%)	90 (46%)	136 (43%)	219 (44%)	.074
Paravertebral involvement	15 (14%)	38 (26%)	127 (64%)	151 (48%)	199 (40%)	<.001
Psoas involvement	6 (6%)	21 (15%)	44 (22%)	77 (25%)	108 (22%)	<.001
Concurrent infections	14 (13%)	31 (22%)	48 (24%)	95 (30%)	115 (23%)	.058
Endocarditis	6 (6%)	13 (9%)	23 (12%)	42 (13%)	57 (12%)	.091
Blood culture obtained	99 (93%)	133 (92%)	187 (95%)	300 (96%)	461 (93%)	.889
Bone biopsy performed	53 (50%)	56 (39%)	69 (35%)	108 (34%)	102 (21%)	<.001
Blood culture positive (N = 1180)	53 (54%)	87 (65%)	132 (71%)	208 (69%)	279 (61%)	.996
Tissue culture positive (N = 388)	29 (55%)	35 (62%)	35 (51%)	70 (65%)	72 (71%)	.033
Final pathogen positive	78 (74%)	118 (82%)	152 (77%)	256 (82%)	357 (72%)	.148
Pathogen (N = 961) [not mutually exclusive]						
MSSA	39 (50%)	37 (31%)	61 (40%)	93 (36%)	121 (34%)	.077
MRSA	13 (17%)	19 (16%)	19 (12%)	36 (14%)	36 (10%)	.052
Streptococci	6 (8%)	35 (30%)	36 (24%)	68 (27%)	70 (20%)	.752
Gram-negative bacilli	5 (6%)	10 (8%)	18 (12%)	22 (9%)	49 (14%)	.048
Enterococci	3 (4%)	13 (11%)	11 (7%)	18 (6%)	31 (9%)	.582
Other	12 (15%)	10 (8%)	17 (11%)	34 (13%)	60 (17%)	.088
Intravenous antibiotic use	100 (94%)	138 (96%)	192 (97%)	309 (98%)	467 (95%)	.651
Duration of IV antibiotics, d (N = 1206)	42 (42–56)	42 (42–56)	42 (42–66)	46 (42–69)	42 (42–61)	.045
Oral antibiotic use	32 (30%)	37 (26%)	79 (40%)	127 (40%)	251 (51%)	<.001
Duration of PO antibiotics, d (N = 526)	70 (37–552)	365 (57–1964)	69 (28–278)	104 (30–573)	180 (44–700)	.082
Duration of total antibiotics, d	56 (42–82)	42 (42–84)	63 (42–106)	58 (42–120)	74 (42–246)	<.001
Initial surgical intervention performed	14 (13%)	21 (15%)	25 (13%)	46 (15%)	58 (12%)	.496
Treatment Failure, 1-y rate	16% (8%–23%)	14% (8%–20%)	14% (9%–18%)	12% (9%–16%)	10% (7%–12%)	.045

Abbreviations: CRP, C-reactive protein; ESR, erythrocyte sedimentation rate; IV, intravenous; MRSA, methicillin-resistant *Staphylococcus aureus*; MSSA, methicillin-susceptible *Staphylococcus aureus*; PO, per os (oral); WBC, white blood cell count.

Comorbidity burden increased significantly, reflected by increasing CCI (median [IQR] 3 [1–5] to 4 [2–7]; *P* < .001) and increasing prevalence of CKD (10% to 21%; *P* < .001), active chemotherapy (6% to 11%; *P* < .001), and immunosuppressive conditions (8% to 17%; *P* = .010). Paravertebral and psoas involvement increased through 2010–2014, while bone biopsy rates declined steadily (50% to 21%; *P* < .001). Microbiological profiles shifted modestly, with decreasing MRSA prevalence (17% to 10%; *P* = .052) and increasing Gram-negative bacill (GNB) (6% to 14%; *P* = .048). One-year treatment failure rates declined over time (16% to 10%; *P* = .045).

### Predictors of Treatment Failure and Mortality

Multivariable analysis using competing risk regression, showed that diabetes mellitus (subdistribution hazard ratio [sHR] 1.92, 95% confidence interval [CI] 1.18–3.13) and multilevel spinal involvement (sHR 1.67, 95% CI 1.17–2.38) were independently associated with increased treatment failure, whereas concurrent infections (sHR 0.57, 95% CI 0.37–0.87) and higher CCI (sHR 0.62, 95% CI 0.43–0.90) were associated with decreased failure risk. In multivariable survival analysis, treatment failure independently predicted subsequent mortality (HR 1.46, 95% CI 1.08–1.96). Other independent predictors of death included older age (HR 2.04, 95% CI 1.57–2.66), higher CCI (HR 1.79, 95% CI 1.37–2.35), hemodialysis (HR 3.62, 95% CI 2.02–6.49), diabetes mellitus (HR 1.29, 95% CI 1.01–1.65), and concurrent infections (HR 1.50, 95% CI 1.20–1.89). Surgical intervention was independently associated with reduced mortality risk (HR 0.61, 95% CI 0.43–0.86) (Table 7).

**Table 7.** Independent Effects on Treatment Failure and Death

	Treatment Failure^b^		Death^b^	
Predictor	sHR^c^ (95% CI)	*P*	HR^c^ (95% CI)	*P*
Age	0.96 (0.77–1.19)	.723	2.04 (1.57–2.66)	<.001
Male sex	1.01 (0.72–1.40)	.975	1.14 (0.93–1.40)	.217
Diabetes mellitus	1.92 (1.18–3.13)	.009	1.29 (1.01–1.65)	.041
Charlson Comorbidity Index	0.62 (0.43–0.90)	.012	1.79 (1.37–2.35)	<.001
Hemodialysis	1.04 (0.25–4.25)	.959	3.62 (2.02–6.49)	<.001
Immunosuppressive condition	0.93 (0.56–1.53)	.766	1.17 (0.90–1.54)	.245
Concurrent infections	0.57 (0.37–0.87)	.009	1.50 (1.20–1.89)	<.001
Multilevel involvement	1.67 (1.17–2.38)	.005	1.09 (0.86–1.39)	.463
Spinal-associated involvement^a^	0.79 (0.57–1.11)	.178	1.15 (0.94–1.41)	.181
Pathogen	…	.142	…	.234
MSSA	1.06 (0.68–1.64)	…	0.92 (0.70–1.23)	…
MRSA	1.55 (0.90–2.67)	…	1.16 (0.82–1.65)	…
Streptococci	0.66 (0.36–1.19)	…	0.82 (0.60–1.12)	…
Other	0.98 (0.62–1.56)	…	0.82 (0.61–1.10)	…
Not identified	1.00	…	1.00	…
Antibiotic duration^d^	1.21 (0.98–1.49)	.118	1.04 (0.82–1.33)	.189
Surgical intervention^d^	1.19 (0.77–1.82)	.432	0.61 (0.43–0.86)	.005
Treatment failure^d^	…	…	1.46 (1.08–1.96)	.013

Abbreviations: CI, confidence interval; MRSA, methicillin-resistant *Staphylococcus aureus*; MSSA, methicillin-susceptible *Staphylococcus aureus*; sHR, subdistribution hazard ratio.

^a^Spinal-associated involvement was defined as the presence of epidural, paravertebral, or psoas involvement.

^b^Time to treatment failure and time to death were analyzed, respectively, using extended proportional subdistribution hazards regression (with death treated as a competing risk) and extended proportional hazards regression models allowing for time-dependent covariates. The table lists the 12 hypothesized risk factors included in both of the multivariable models, plus the additional covariate for treatment failure included in the mortality model.

^c^The adjusted sHR for treatment failure and the adjusted HR for death represent the independent effect of the selected predictor variable on the risk of the corresponding endpoint, controlling for all other variables in the model. The sHR and HR for continuous predictors were calculated comparing the third with the first quartiles (ie, age, 75.4 : 56.9 y; CCI, 3 : 0; antibiotic duration, 108 : 42 d).

^d^Predictor was incorporated into the model as a time-dependent variable.

## DISCUSSION

Despite increasing global recognition and rising incidence, the clinical characteristics and long-term outcomes of NVO remain incompletely understood, with limited large-scale data on predictors of treatment failure. To our knowledge, this is the largest NVO cohort, providing a comprehensive overview of its current clinical landscape. We observed the growing prevalence of comorbidities, particularly CKD, malignancy, and immunosuppression, which underscores the increasing complexity of NVO and the need for tailored management in high-risk population.

The lumbar spine was most frequently involved, with epidural or paravertebral extension seen in over 40% of patients. Concurrent infections, including endocarditis, were also frequent, emphasizing the hematogenous nature of NVO and the importance of thorough systemic evaluation [13, 22, 23].

Accurate microbiologic diagnosis remains the cornerstone but continues to pose substantial challenges. Blood cultures identified the pathogens in nearly two-thirds of cases, underscoring their importance as the initial diagnostic step. Bone biopsy added diagnostic yield, and combined blood and tissue cultures reached 76% positivity. The addition of broad range PCR modestly improved pathogen detection, particularly among culture-negative cases, where two-thirds of PCR-positive results occurred. These findings illustrate the complementary roles of culture-based and molecular methods and support a multimodal approach integrating blood cultures, image-guided or intraoperative biopsy, and selective molecular testing. Lessons from our institutional experience suggest that while molecular testing enhances diagnostic confidence in difficult-to-culture infections, its use should be selective and guided by clinical context, given cost, and turnaround time.

Importantly, microbiological patterns shifted over time: while MSSA remained the most common pathogen [7, 12, 14, 18, 19, 24], MRSA decreased, and GNB increased. These shifts likely reflect evolving host–pathogen dynamics and antimicrobial exposures, reinforcing the need for careful empiric selection and consideration of potential portals of entry.

Most patients received 6–8 weeks of IV antibiotics, consistent with the IDSA guideline [3] and the subsequent observational cohort [12]. Total antibiotic duration increased over time, largely driven by expanded use of oral step-down regimens, despite evidence from an RCT demonstrating non-inferiority of shorter courses [17]. This likely reflects cautious practice, with treatment individualized by comorbidities, infection severity, concurrent infections, and treatment response. Although the Oral versus Intravenous Antibiotics for Bone and Joint Infection (OVIVA) trial demonstrated non-inferiority of early oral therapy for bone and joint infections [25] and recent trends in the management of musculoskeletal infections supporting a shift from IV to oral therapy [25–29], the median IV duration in our cohort remained approximately 6 weeks, and early oral switch (<2 weeks) remained uncommon. This aligns with a recent U.S. survey identifying NVO as one of the most challenging musculoskeletal infections for implementing oral therapy [28]. Our prior meta-analysis similarly found no increased failure with early oral switch, though available studies were limited by high risk of bias [30]. Because oral step-down timing in our cohort was clinician-driven and time-dependent, precluding valid comparison with all-IV regimens; therefore, our study aimed to describe management patterns rather than compare outcomes. These findings highlight the need for NVO-specific trials evaluating oral therapy. Although a small number of fungal and mycobacterial infections were included in this cohort (approximately 5% of cases), these represented a minor proportion and are unlikely to have affected the overall results. Their inclusion reflects our intent to capture the full clinical and microbiological spectrum of NVO encountered in routine practice.

The cumulative treatment failure rate reached 14% after 7 years, comparable to prior large studies (13%–19%) [12, 18], whereas smaller cohorts have shown broader variability (13%–48%) due to differences in study design, follow-up duration, and outcome definitions [13, 14, 19, 24]. In our cohort, failures tended to occur early (median 3.1 months), consistent with our prior experience [7], emphasizing the importance of close monitoring in the early phase. Notably, despite increasing baseline risk, 1-year failure rates declined over time, suggesting improved recognition, diagnostics, and individualized care. These patterns merit further exploration in prospective studies.

The association between diabetes mellitus and treatment failure aligns with previous studies, potentially due to the negative effects of hyperglycemia on immune function, vascularization, and tissue healing. Hyperglycemia promotes bacterial growth and biofilm formation [31] and impairs neutrophil migration, phagocytosis, and oxidative burst [32]. Diabetes-related microvascular complications may further impair tissue perfusion and oxygenation, delaying healing and increasing recurrence risk [33, 34]. These mechanisms underscore the importance of glycemic control in managing osteomyelitis in diabetic patients.

Similarly, the association between multilevel vertebral involvement and treatment failure may be explained by several hypotheses. First, it may reflect more aggressive pathogens and higher bacterial burden, contributing to persistent or recurrent infection. Second, delayed diagnosis may allow progression and more extensive bony destruction, complicating source control [35, 36]. Third, the anatomical complexity of multi-level disease may impair antimicrobial penetration, particularly in avascular regions, limiting therapeutic efficacy [37].

Although not statistically significant in our multivariable model, MRSA showed a trend toward increased treatment failure. This aligns with previous studies reporting high recurrence rates [12, 18, 22], possibly due to substantial comorbidities, limited antibiotic options, subtherapeutic vancomycin levels [22], poor bone penetration [37], and biofilm formation even without implants [38, 39]. Thus, MRSA remains clinically important despite the lack of statistical significance.

Both higher CCI and concurrent infections were unexpectedly associated with reduced failure, despite our attempts to account for attrition by death via the use of competing risk analysis. The inverse association with higher CCI may reflect residual survivor bias, as highly comorbid patients had greater mortality before experiencing failure, or may relate to closer monitoring and more prolonged or intensive therapy. Post hoc analysis showed patients with concurrent infections received significantly longer antibiotic courses (median 75 vs 56 days, *P* = .008) (Supplementary Table 1). However, a prior RCT found no benefit of prolonged therapy [12], suggesting that factors beyond treatment duration may contribute to this association. Prospective studies are warranted to clarify these associations and guide management for high-risk subgroups.

This study has several limitations. First, it is retrospective in nature and subject to inherent biases, including unmeasured confounding, selection bias, and missing data. Second, while we included only patients with MRI findings consistent with NVO, this selection criteria may have excluded patients who were unable to undergo MRI due to contraindications, intolerance, or clinical instability. Nonetheless, MRI remains the most recommended and accessible modality [40]. Third, treatment strategies—including antibiotic selection, duration, and timing of surgery—were not protocolized and likely varied across sites, providers, and time periods. Fourth, although we observed a substantial increase in NVO cases during the study period, especially over the last decade, population-based and hospital-based denominators were unavailable. Therefore, it remains unclear whether this observation reflects heightened awareness, improved diagnostics, or a true increase in disease burden among aging and immunocompromised hosts [11]. Fifth, details regarding biopsy site (vertebral body vs intervertebral disc) and number of specimens were not consistently available across participating centers throughout the study period, limiting the granularity of diagnostic performance analysis. Finally, we did not capture standardized functional or quality-of-life outcomes, which may underestimate the long-term burden of disability or patient-centered outcomes.

In summary, this 26-year multicenter cohort study provides the most comprehensive long-term evaluation of NVO to date, demonstrating clinical complexity. Diabetes mellitus and multilevel involvement independently predicted treatment failure, mostly within the first 6 months. These findings highlight the importance of early risk stratification and tailored management through coordinated multidisciplinary follow-up involving orthopedic surgery, infectious diseases, outpatient parenteral antimicrobial therapy, and pharmacy teams during the first few months. Close monitoring of high-risk patients allows timely adjustment of antimicrobial therapy, surgical reassessment, and repeat imaging as needed when clinical or laboratory response is suboptimal. Future research should investigate the role of modern diagnostics and evolving treatment paradigms in optimizing outcomes for this complex and increasingly prevalent infection.

## Supplementary Material

ciag048_Supplementary_Data

## Supplementary Data

Supplementary materials are available at *Clinical Infectious Diseases* online. Consisting of data provided by the authors to benefit the reader, the posted materials are not copyedited and are the sole responsibility of the authors, so questions or comments should be addressed to the corresponding author.

## Note

***Financial support*.** This project was supported by internal funding from the Mayo Clinic Division of Public Health, Infectious Diseases and Occupational Medicine, which supported statistical analysis.
