# Supplementary material for: Clinical Manifestations, Long-Term Trends, and Risk Factors for Treatment Failure in Native Vertebral Osteomyelitis: A 26-Year Mayo Clinic Experience
Source: Clin Infect Dis. 2026 Feb 4;82(6):e1171–80. doi: 10.1093/cid/ciag048 (PMC13341251; doi:10.1093/cid/ciag048)
Supplement: ciag048_Supplementary_Data [file ciag048_supplementary_data.docx]

**Supplementary Methods 1.**

Using a search strategy based on radiology reports that enabled the construction of a long-term cohort, we queried MRI reports with the following terms: (discitis) OR (spondylitis) OR (spondylodiscitis) OR (vertebral osteomyelitis) OR (diskitis) OR (spondylodiskitis) OR (disc space infection) OR (disk space infection) OR (vertebral infection) OR (spinal infection) OR (intervertebral infection) OR (discovertebral infection) OR (diskovertebral infection) [1].

Reference

1. Petri F, Mahmoud O, El Zein S, et al. It is time for a unified definition of native vertebral osteomyelitis: a framework proposal. J Bone Jt Infect **2024**; 9:173–182.

**Supplementary Methods 2.**

Baseline characteristics were described by medians and interquartiles for continuous variables and by frequencies and percentages for discrete variables. Outcomes were assessed by treatment failure and all-cause mortality, and the occurrence of each was described by cumulative event rates as a function of time after NVO diagnosis. Specifically, a nonparametric cumulative incidence function estimator under competing risks was used to compute rates of treatment failure (accounting for the competing risk of mortality), while the Kaplan-Meier survival estimator was used to compute cumulative mortality. Patient characteristics were further described over our 26-year experience according to five different time periods, and temporal changes in the patient characteristics were evaluated using the monotonic trend (Spearman) test for continuous variables, the Mantel-Haenszel trend test for discrete variables, and a test for linear trend based on a Fine-Gray competing risk model for treatment failure. All analyses were conducted in R version 4.4.1 (R Core Team, 2024) and used 0.05-level significance tests.

To elucidate the clinical factors that influence treatment failure, we performed a multivariable competing risks analysis based on the subdistribution hazard regression approach of Fine and Gray, with failure modeled as the primary event of interest and death treated as the competing event. The model included prespecified potential predictors of age, sex, diabetes mellitus, Charlson comorbidity index (excluding age weights from the scoring), end stage renal disease, immunosuppressed status, concurrent infections, multi-level involvement, spinal-associated abscess, pathogen, and time-dependent variables for antibiotic duration (therapy lasted a median of 59 days but varied considerably) and surgical intervention (usually within a few days of diagnosis). These potential predictors were entered in the model without variable selection, which was fitted using an extended form of the subdistribution hazard model to incorporate the time-dependent covariates. The clinical determinants of mortality were analyzed in similar multivariable fashion with extended Cox proportional hazards regression, without using treatment failure as a competing event but rather including it as an additional time-dependent covariate in the model. The time-dependent antibiotic duration variable was coded as 0 until the start of treatment, then incrementally adding 1 for each day on antibiotics, cumulatively up to the last treatment day and carried forward thereafter. By default, continuous predictors were modeled without assuming linearity using restricted cubic splines. However, those variables for which the spline fits to the data provided little to no evidence of nonlinearity were re-fitted in the final model assuming linearity.

**Supplementary Table 1. Post Hoc Comparison of Baseline Characteristics, Diagnostic Workup, and Treatment Between Patients With and Without Concurrent Infections**

|  | | **Concurrent Infections** | |  |
| --- | --- | --- | --- | --- |
| **Variable** | **N** | **No (N=952)** | **Yes (N=303)** | **P** |
| Age, years | 1255 | 67 (56-75) | 67 (58-75) | 0.680^1^ |
| Male sex | 1255 | 599 (63%) | 229 (76%) | < 0.001^2^ |
| Body Mass Index, kg/m² | 1214 | 28 (24-34) | 29 (26-33) | 0.065^1^ |
| Race: White | 1213 | 858 (93%) | 274 (93%) | 0.921^2^ |
| Diabetes mellitus | 1255 | 207 (22%) | 58 (19%) | 0.334^2^ |
| Chronic kidney disease | 1255 | 166 (17%) | 43 (14%) | 0.187^2^ |
| Hemodialysis | 1255 | 19 (2%) | 1 (0%) | 0.044^2^ |
| Cancer history | 1255 | 160 (17%) | 36 (12%) | 0.040^2^ |
| Active chemotherapy | 1255 | 79 (8%) | 15 (5%) | 0.054^2^ |
| Charlson Comorbidity Index | 1255 | 3 (2-6) | 3 (2-5) | 0.307^1^ |
| IV drug user | 1255 | 16 (2%) | 7 (2%) | 0.477^2^ |
| History of any transplant | 1255 | 32 (3%) | 8 (3%) | 0.534^2^ |
| Immunosuppressive condition | 1255 | 151 (16%) | 32 (11%) | 0.023^2^ |
| Back pain | 1255 | 846 (89%) | 269 (89%) | 0.967^2^ |
| Fever | 1255 | 469 (49%) | 202 (67%) | < 0.001^2^ |
| Motor weakness | 1255 | 352 (37%) | 130 (43%) | 0.065^2^ |
| Numbness | 1255 | 262 (28%) | 79 (26%) | 0.622^2^ |
| WBC at diagnosis | 1123 | 9 (6-12) | 10 (8-14) | < 0.001^1^ |
| CRP at diagnosis | 979 | 45 (13-112) | 113 (35-198) | < 0.001^1^ |
| ESR at diagnosis | 837 | 56 (31-88) | 67 (46-98) | < 0.001^1^ |
| Cervical involvement | 1255 | 104 (11%) | 53 (17%) | 0.003^2^ |
| Thoracic involvement | 1255 | 366 (38%) | 113 (37%) | 0.719^2^ |
| Lumbosacral involvement | 1255 | 602 (63%) | 220 (73%) | 0.003^2^ |
| Multi-level involvement | 1255 | 181 (19%) | 77 (25%) | 0.016^2^ |
| Epidural involvement | 1255 | 413 (43%) | 123 (41%) | 0.393^2^ |
| Paravertebral involvement | 1255 | 390 (41%) | 140 (46%) | 0.108^2^ |
| Psoas involvement | 1255 | 172 (18%) | 84 (28%) | < 0.001^2^ |
| Endocarditis | 1255 | 0 (0%) | 141 (47%) | < 0.001^2^ |
| Blood culture obtained | 1255 | 889 (93%) | 291 (96%) | 0.089^2^ |
| Bone biopsy performed | 1255 | 352 (37%) | 36 (12%) | < 0.001^2^ |
| Broad range PCR performed | 1255 | 131 (14%) | 27 (9%) | 0.027^2^ |
| Blood culture positive | 1180 | 499 (56%) | 260 (89%) | < 0.001^2^ |
| Tissue culture positive | 388 | 217 (62%) | 24 (67%) | 0.554^2^ |
| Broad-range PCR positive | 158 | 39 (30%) | 10 (37%) | 0.457^2^ |
| Final pathogen positive | 1255 | 680 (71%) | 281 (93%) | < 0.001^2^ |
| Pathogen | 961 |  |  | < 0.001^2^ |
| MSSA |  | 246 (36%) | 104 (37%) |  |
| MRSA |  | 80 (12%) | 43 (15%) |  |
| Streptococci |  | 133 (20%) | 77 (27%) |  |
| Gram-negative |  | 84 (12%) | 10 (4%) |  |
| Enterococci |  | 37 (5%) | 27 (10%) |  |
| Other |  | 100 (15%) | 20 (7%) |  |
| Intravenous antibiotic use | 1255 | 910 (96%) | 296 (98%) | 0.100^2^ |
| Duration of IV antibiotics, days | 1206 | 42 (42-59) | 44 (42-68) | 0.474^1^ |
| Oral antibiotic use | 1255 | 389 (41%) | 137 (45%) | 0.181^2^ |
| Duration of PO antibiotics, days | 526 | 90 (39-512) | 365 (77-1042) | < 0.001^1^ |
| Duration of total antibiotics, days | 1255 | 56 (42-112) | 75 (42-280) | 0.008^1^ |
| Surgical intervention performed | 1255 | 126 (13%) | 38 (13%) | 0.755^2^ |
| Time to surgery, days | 164 | 1 (0-5) | 2 (1-6) | 0.688^1^ |

Data are presented as median (25th percentile-75th percentile) for continuous variables and number (percentage) of patients for categorical variables. N represents the number of non-missing values. Tests used: ^1^Wilcoxon rank sum test; ^2^Pearson χ² test

Abbreviations: BMI, Body Mass Index; CCI, Charlson Comorbidity Index; IV, Intravenous; ESRD, End-Stage Renal Disease; CRP, C-reactive protein; ESR, Erythrocyte Sedimentation Rate; WBC, White Blood Cell count; PCR, Polymerase Chain Reaction; MSSA, Methicillin-Susceptible *Staphylococcus aureus*; MRSA, Methicillin-Resistant *Staphylococcus aureus*; PO, per os (oral).
